# Head-Specific Spatial Spectra of Electroencephalography Explained: A Sphara and BEM Investigation

**DOI:** 10.3390/bios15090585

**Published:** 2025-09-06

**Authors:** Uwe Graichen, Sascha Klee, Patrique Fiedler, Lydia Hofmann, Jens Haueisen

**Affiliations:** 1 Division Biostatistics and Data Science, Karl Landsteiner University of Health Sciences, Dr.-Karl-Dorrek-Str. 30, 3500 Krems an der Donau, Austria; sascha.klee@kl.ac.at; 2 Institute of Biomedical Engineering and Informatics (BMTI), Faculty of Computer Science and Automation, Technische Universität Ilmenau, Gustav-Kirchhoff-Str. 2, 98693 Ilmenau, Germany; patrique.fiedler@tu-ilmenau.de (P.F.); lydia.hofmann@tu-ilmenau.de (L.H.); 3 Biomagnetic Center, Department of Neurology, University Clinic Jena, Erlanger Allee 101, 07747 Jena, Germany

**Keywords:** spatial Nyquist theorem, spatial filtering, Boundary Element Method (BEM), volume conductor modeling, spatial harmonic analysis (Sphara), Electroencephalography (EEG), frequency response, transcranial electrical stimulation (tES), forward modeling, head model

## Abstract

Electroencephalography (EEG) is a non-invasive biosensing platform with a spatial-frequency content that is of significant relevance for a multitude of aspects in the neurosciences, ranging from optimal spatial sampling of the EEG to the design of spatial filters and source reconstruction. In the past, simplified spherical head models had to be used for this analysis. We propose a method for spatial frequency analysis in EEG for realistically shaped volume conductors, and we exemplify our method with a five-compartment Boundary Element Method (BEM) model of the head. We employ the recently developed technique for spatial harmonic analysis (Sphara), which allows for spatial Fourier analysis on arbitrarily shaped surfaces in space. We first validate and compare Sphara with the established method for spatial Fourier analysis on spherical surfaces, discrete spherical harmonics, using a spherical volume conductor. We provide uncertainty limits for Sphara. We derive relationships between the signal-to-noise ratio (SNR) and the required spatial sampling of the EEG. Our results demonstrate that conventional 10–20 sampling might misestimate EEG power by up to 50%, and even 64 electrodes might misestimate EEG power by up to 15%. Our results also provide insights into the targeting problem of transcranial electric stimulation.

## 1. Introduction

It is known that the human head, considered as a volume conductor, possess spatial low-pass filter characteristics [1,2,3,4,5,6]. An exact analysis and quantification of these low-pass filter characteristics of the human head is helpful for the interpretation of the potential distribution on the scalp, the design and interpretation of the results of source reconstruction methods, the estimation of the minimum necessary number of sensors for spatial sampling, the design of spatial filter methods and spatial compressed sensing approaches in EEG systems, as well as the compressed representation of the measured EEG data.

An approach that has been used so far to quantify the low-pass filter characteristics of the head is based on a combination of spherical head models to model the volume conduction properties and spherical harmonics (SH) [1,2,3,4]. However, spherical head models are only a rough approximation of the real properties of the head. A mapping of the real electrode positions on the head surface to a spherical surface is necessary, which leads to mapping errors. For anatomical reasons, not all areas of the head around the brain can be sampled, e.g., areas of the neck and the nasal cavity. These areas must be excluded using a window function. The error caused by the mapping and the window function is difficult to estimate.

In another approach, EEG was measured on the scalp with curvilinear arranged electrodes [5]. In a similar approach, the spatial spectra of the cortex surface and the potential distribution were compared in a simulation study. The analysis was performed on one-dimensional contours [6]. In the latter two cases, spatially spectral analysis was carried out via 1D discrete Fourier transform. In this way, only the one-dimensional aspects of the higher-dimensional problem are taken into account. In addition, the measured or simulated data are also mapped to another domain in order to analyze them, which also leads to errors that are difficult to estimate.

Evidence supporting the spatially band-limited nature of bioelectric and biomagnetic fields has recently been reported by Iivanainen et al., who introduced a generalized spatial-frequency framework combined with optimal sensor layout design [7]. Simulations involving a realistic adult head demonstrated that almost all informative scalp EEG content could be captured using approximately 110 electrodes, thereby placing an upper limit on the advantages of further sensor densification.

Recent work also highlights the dual importance of sensor layout and geometry-aware spectral bases. By systematically down-sampling high-density infant EEG, Asayesh et al. (2024) demonstrated that global network measures reach a plateau at around 60 electrodes, whereas fine-scale patterns continue to benefit from arrays of 128 channels or more [8]. In addition, Giri et al. (2022) proposed a ‘realistic four-shell head’ adapted ‘anatomical harmonics’ basis and demonstrated substantial gains in inverse-problem speed and accuracy without compromising localization precision [9].

In parallel, recent work has explored data-driven strategies to enhance the apparent spatial resolution of EEG; for example, using Transformer-based super-resolution [10] and diffusion-based generative models [11]. While these approaches are thematically distinct from the present study, our quantitative analysis of the head’s intrinsic spatial-frequency response provides a biophysical reference that can help constrain or regularize such learned reconstructions, ensuring their outputs remain consistent with the physical limits of scalp potentials.

A new approach for spatial harmonic analysis (Sphara) was introduced that extends the classical spatial Fourier analysis to systems with non-uniformly positioned sensors, such as EEG sensor systems [12]. Sphara is based on the eigenanalysis of the discrete Laplace–Beltrami operator defined on a triangular mesh, which is formed based on the sensor positions. Sphara allows a spatial Fourier analysis of the spatial potential distribution of EEG data. Thus, the spatial low-pass filter characteristics can be examined using realistic head models in a convenient way. Beyond this methodological foundation, Sphara has already proven its usefulness in practical applications; for example, in the comparison of different EEG sensor technologies on the same head geometry [13].

In the following sections of this paper, we will briefly outline the Sphara approach and discuss its essential properties. In order to validate the suitability of Sphara for the analysis of the spatial frequency response of volume conductors and to assess the uncertainties of Sphara in quantitative analyses, we compare the results of the discrete spherical harmonics and Sphara decomposition using spherical head models. Subsequently, we extend the analysis from idealized spherical head models to realistic anatomy by employing the Boundary Element Method (BEM) to compute scalp potentials on a standard realistic head model (see Section 3.2).

## 2. Materials and Methods

### 2.1. Generalized Spatial Fourier Analysis—Sphara

Sphara extends the classical spatial Fourier analysis to sensors positioned non-uniformly on a surface [12]. In practical applications, surfaces are often represented by triangular meshes. For the EEG, parts of the head surface can be triangulated using the electrode positions. A generalized spatial Fourier basis can be computed via eigenanalysis of the discrete Laplace–Beltrami operator, which is defined on such a triangle mesh. Thus, the Sphara basis functions, which are used in the presented approach for spatial Fourier analysis, are determined adaptively for an underlying spatial sampling scheme.

A triangular mesh M={V,E,T} is used to represent the sensor layout, with vertices v∈V (sensor positions), edges e∈E, and triangles t∈T (cf. [12]). The EEG potential distribution is discretely sampled at the vertices (sensor positions) and stored in a vector $\vec{f}$. The discrete Laplace–Beltrami operator is then applied in matrix form as

(1) $$\Delta_{D}\vec{f}=-L\vec{f}\;,$$

where *L* is a Laplacian matrix of size n×n, with n=|V|.

Following the finite element method (FEM), *L* is expressed as $L=B^{-1}S$, with stiffness matrix *S* and mass matrix *B* [12,14,15,16,17,18]. The FEM formulation with piecewise linear basis functions yields a generalized eigenproblem

(2) $$S{\vec{x}}_{i}=\tau_{i}B{\vec{x}}_{i}\;,$$

whose eigenvectors ${\vec{x}}_{i}$ form the spatial Fourier basis. Orthogonality is ensured with respect to the inner product related to the *B* matrix,

(3) $${\langle \vec{f},{\vec{x}}_{i}\rangle }_{B}={\vec{f}}^{\;⊤}B{\vec{x}}_{i}\;,$$

and eigenvectors are normalized accordingly.

The corresponding eigenvalues $\tau_{i}$ with $\tau_{i}\in R$ and $\tau_{i}\geq 0$ can be used to determine the spatial wavenumber $k_{i}$, spatial frequency $\omega_{i}$, and consequently, the spatial wavelength $\lambda_{i}$:

(4) $$k_{i}=\sqrt{\tau_{i}}=2\pi \omega_{i}=\frac{2\pi }{\lambda_{i}}\;.$$

**Figure 1:**
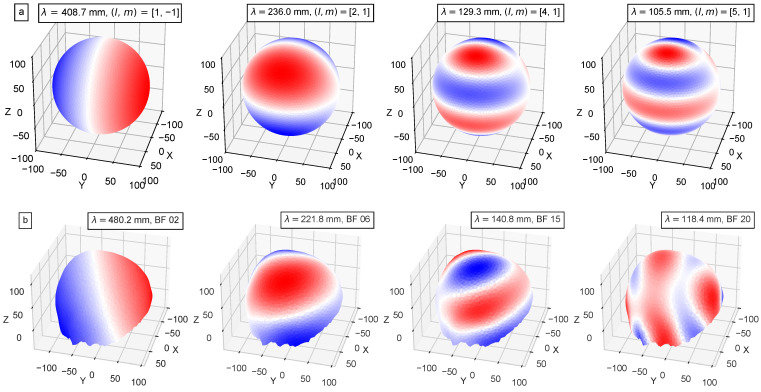
A selection of spherical harmonic and Sphara BF: For each BF, the corresponding spatial wavelength is given. Red and blue indicate positive and negative values of the function, respectively, with the colour intensity reflecting the magnitude. (**a**) In the upper row, some SH BF are shown with degree *l* and order *m*. (**b**) In the lower row, some Sphara BF are displayed, determined for the head surface covered by the EEG sensor system.

In this paper, we use λ to denote the wavelength; thus, τ denotes the eigenvalues. A selection of Sphara BF calculated using the FEM discretization of the Laplace–Beltrami operator can be seen in the bottom row of Figure 1. A more detailed derivation of the Sphara method to compute the generalized spatial Fourier basis can be found in [12]. We performed the Sphara analysis using the Python toolbox SpharaPy (version 1.1.2) [19].

### 2.2. Spherical Harmonic Analysis

Spherical harmonics are functions that are defined on the surface of a sphere. These functions form a basis that can be used for Fourier analysis on spherical surfaces. The real form of the spatial harmonics is defined as follows:

(5) $$Y_{lm}\left(\Omega \right)=\left\{\begin{array}{cc} {\bar{P}}_{l}^{m}\left(\cos(\theta )\right)\;\cos\left(m\;\phi \right) & \mathrm{if}\;m\geq 0\\ {\bar{P}}_{l}^{\left|m\right|}\left(\cos(\theta )\right)\;\sin\left(|m|\;\phi \right) & \mathrm{if}\;m<0 \end{array}\right.\;,$$

with the domain Ω=(θ,ϕ), θ and ϕ are colatitude and longitude, respectively, and *l* and *m* are the spherical harmonic degree and order, respectively. ${\bar{P}}_{l}^{m}$ are normalized associated Legendre functions

(6) $${\bar{P}}_{l}^{m}\left(x\right)=\sqrt{\left(2-\delta_{0m}\right)\left(2l+1\right)\frac{(l-m)!}{(l+m)!}}P_{l}^{m}\left(x\right)\;,$$

with the associated Legendre function $P_{l}^{m}$ and the Kronecker delta function $\delta_{ij}$. The spherical harmonics analysis and synthesis can be performed using the following equations:

(7) $$F_{lm}=\frac{1}{4\pi }\int_{\Omega }f\left(\Omega \right)\;Y_{lm}\left(\Omega \right)d\Omega$$

and

(8) $$f\left(\Omega \right)=\sum_{l=0}^{\infty }\;\sum_{m=-l}^{l}F_{lm}\;Y_{lm}\left(\Omega \right)\;.$$

The equivalent Cartesian wavelength of spherical harmonics of degree *l*, defined on a sphere with radius *R*, is determined by the Jeans relation [20]:

(9) $$\lambda =\frac{2\;\pi \;R}{\sqrt{l(l+1)}}\;.$$

A selection of spherical harmonic basis functions is shown in the upper row of Figure 1.

In analogy to the Nyquist–Shannon sampling theorem for temporal signals [21], sufficient sampling must be ensured if signal components up to a certain spatial frequency are to be registered. For spherical harmonics, the spatial frequency is determined by the degree *l*, c.f. Equation (9). Various sampling schemes have been presented in literature, which allow for a resolution up to a given degree *l* of the discrete spherical harmonics [20,22,23]. One sampling scheme is based on the Gauss–Legendre quadrature (GLQ), where the sampling points are arranged on a grid of size

(10) $$N_{GLQ}=\left(l+1\right)\times \left(2l+1\right)$$

in the latitude and longitude direction [20,22]. Another scheme introduced by Driscoll and Healy (DH) allows for sampling on a regular grid using the following sampling points [20,23]:

(11) $$N_{DH}=\left(2l+2\right)\times \left(2l+2\right)$$

These sampling schemes are angle-based in longitude and co-latitude. This results in a different density of spatial sampling points in different areas of the sphere surface. They are particularly dense near 0° and 180° co-latitude, respectively. Thus, for technical reasons, sampling schemes similar to these cannot be implemented in EEG systems with higher sensor numbers. Therefore, we have used an equidistant sampling scheme in our simulations with head models based on concentric spherical shells. An upper bound $l_{up}$ for the degree of discrete spherical harmonics that can be resolved with *n* spatial sampling points can be estimated as follows:

(12) $$l_{up}=-1+\left\lfloor \frac{1}{2}\sqrt{n}\right\rfloor \;.$$

The discrete spherical harmonics coefficients of the equidistant sampled data points of the spherical head model are computed via least squares inversion using the SHExpandLSQ routine of the SHTOOLS toolbox [20].

### 2.3. Metrics

The discrete spherical harmonics and the Sphara transform can be considered as a generalized spatial Fourier transform for the investigated domains. Both transformations are unitary. Therefore, the best approximation in terms of least squares (${𝓁}_{2}$ norm) in the spatial frequency domain is also the best approximation in the spatial domain (cf. [24] Section 3.4). Consequently, ${𝓁}_{2}$ norm-based metrics (e.g., energy and power measures) are applied and compared within and between both domains.

### 2.4. Spatial Nyquist Limit on Triangular Meshes

In analogy to the temporal Nyquist–Shannon sampling theorem [21], a spatial Nyquist limit exists on triangulated surfaces beyond which higher spatial frequencies cannot be reliably represented. For irregular meshes, a practical and conservative estimate of this limit is based on the maximum edge length $h_{\max}$ of the mesh. Specifically, the spatial Nyquist wavenumber $k_{N}$, frequency $\omega_{N}$, and wavelength $\lambda_{N}$ can be approximated as follows:

(13) $$k_{N}=\frac{\pi }{h_{\max}},\;\omega_{N}=\frac{1}{2h_{\max}},\;\lambda_{N}=2h_{\max}.$$

These estimates represent a *worst-case* bound, ensuring that even in the sparsest region of the mesh, no aliasing occurs for frequencies below $\omega_{N}$. While no formal derivation of this limit exists for arbitrary triangulations, this heuristic is commonly adopted in mesh-based spectral analyses [15,16].

## 3. Simulation Setup

We compare the new Sphara approach for spatial Fourier analysis of arbitrarily arranged sensors and the discrete spherical harmonics using a spherical head model. Following this validation of the new method, a realistic head model is analyzed.

### 3.1. Spherical Model

**Figure 2:**
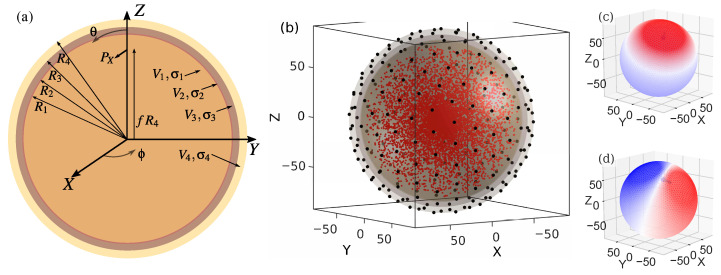
Spherical head model: (**a**) The four-layer head model consists of a layer for the brain, the CSF, the skull, and the skin with the radii $R_{1},\ldots ,R_{4}=$ 80 mm, 81 mm, 86 mm and 92 mm. A single dipole $P_{x}$ aligned in the *x* direction is shown exemplarily. (**b**) Exemplary arrangement of 104 equidistant sensors, black dots. The 7700 sources used for the forward simulation are marked by red dots. The potential distribution on the head surface caused by a radial dipole (**c**) and tangential dipole (**d**). Both dipoles have a distance of 70 mm from the center of the concentric head model. In subfigures (**c**) and (**d**), red and blue indicate positive and negative values of the potential, respectively. The intensity of the colour reflects the magnitude.

The spherical head model consists of four layers, which describe the outer boundary of gray matter $R_{1}$, the inner and outer boundary of the skull (inner skull, outer skull) $R_{2},R_{3}$, and the outer scalp boundary (skin) $R_{4}$, with the corresponding radii of the spherical surfaces $R_{1}=80\;mm$, $R_{2}=81\;mm$, $R_{3}=86\;mm$ and $R_{4}=92\;mm$ [3], see Figure 2.

The four boundary surfaces define the compartments of the brain, cerebrospinal fluid (CSF), skull, and scalp. The following conductivities of the single compartments are used for the simulations: brain and scalp $\sigma_{1}=\sigma_{4}=0.33\;Sm$^−1^, CSF $\sigma_{2}=1.79\;Sm$^−1^, and skull $\sigma_{3}=0.0066\;Sm$^−1^ [25,26,27,28,29].

For a spherical head model, defined by four spherical surfaces, the potential distribution caused by a current dipole in the brain compartment can be semi-analytically determined by a series expansion using Legendre polynomials. Without loss of generality, the potential V of a current dipole, located on the *z*-axis, directed in the *x*-, *y*-, or *z*-direction can be given as follows:

(14) $$\begin{array}{cc} V_{x}\left(\theta ,\phi \right) & =\frac{P_{x}\cos\left(\phi \right)}{4\pi \sigma_{4}R_{4}^{2}}\sum_{l=1}^{\infty }\frac{{\left(2l+1\right)}^{4}f^{\left(l-1\right)}P_{l}^{1}\left(\cos\left(\theta \right)\right)}{l\;C(l)} \end{array}$$

(15) $$\begin{array}{cc} V_{y}\left(\theta ,\phi \right) & =\frac{P_{y}\sin\left(\phi \right)}{4\pi \sigma_{4}R_{4}^{2}}\sum_{l=1}^{\infty }\frac{{\left(2l+1\right)}^{4}f^{\left(l-1\right)}P_{l}^{1}\left(\cos\left(\theta \right)\right)}{l\;C(l)} \end{array}$$

(16) $$\begin{array}{cc} V_{z}\left(\theta ,\phi \right) & =\frac{P_{z}}{4\pi \sigma_{4}R_{4}^{2}}\sum_{l=1}^{\infty }\frac{{\left(2l+1\right)}^{4}f^{\left(l-1\right)}P_{l}\left(\cos\left(\theta \right)\right)}{C(l)}\;, \end{array}$$

where *l* is the number of the term of the series expansion and also the degree of the (associated) Legendre polynomials, $P_{x}$, $P_{y}$, and $P_{z}$ are directed dipoles, respectively, and *f* is the ratio of the distance of the dipole from the center of the model to the radius $R_{4}$ of the outer surface. The two angles θ and ϕ indicate the colatitude and the longitude of a point on the outer surface of the model, cf. Figure 2a. $P_{l}$ and $P_{l}^{1}$ are the Legendre polynomials and the 1st-order associated Legendre polynomials of degree *l*, respectively. The function C(l) for the *l*-th term of the series expansion is defined by the following equation:

(17) $$\begin{array}{cc} C(l)=\; & (\left(lk_{1}+l+1\right)\left(lk_{2}+l+1\right)+\ldots \\ \; & \;+l\left(l+1\right)\left(k_{1}-1\right)\left(k_{2}-1\right){\left(\frac{R_{1}}{R_{2}}\right)}^{\left(2l+1\right)})\;\cdot \;\ldots \\ \; & \;\cdot \;\left(\left(lk_{3}+l+1\right)+\left(l+1\right)\left(k_{3}-1\right){\left(\frac{R_{3}}{R_{4}}\right)}^{\left(2l+1\right)}\right)+\ldots \\ \; & +(\left(k_{1}-1\right)\left(\left(l+1\right)k_{2}+l\right){\left(\frac{R_{1}}{R_{3}}\right)}^{\left(2l+1\right)}+\ldots \\ \; & \;+\left(lk_{1}+l+1\right)\left(k_{2}-1\right){\left(\frac{R_{2}}{R_{3}}\right)}^{\left(2l+1\right)})\;\cdot \;\ldots \\ \; & \;\cdot \;\left(l+1\right)\left(l\left(k_{3}-1\right)+\left(\left(l+1\right)k_{3}+l\right){\left(\frac{R_{3}}{R_{4}}\right)}^{\left(2l+1\right)}\right)\;, \end{array}$$

with the ratios of the conductivities given as $k_{1}=\frac{\sigma_{1}}{\sigma_{2}}$, $k_{2}=\frac{\sigma_{2}}{\sigma_{3}}$ and $k_{3}=\frac{\sigma_{3}}{\sigma_{4}}$ [2,30,31,32]. Dipole positions other than those on the z-axis can be covered by affine transformations of the spherical head model. We have used the FieldTrip toolbox to model the head volume conductor using four concentric spherical shells [31].

The semi-analytical solution using series expansion possesses two useful properties:

1. The *l*-th term of this series contains the *l*-th degree Legendre polynomial $P_{l}$ or the *l*-th degree associated Legendre polynomial $P_{l}^{1}$; compare Equations (14)–(16). By means of the degree *l* and the radius $R_{4}$ of the outer spherical surface, one can determine the spatial wavelength with respect to the spatial frequency of the *l*-th term of the series expansion, to which this term contributes exclusively; see also Equation (9).
2. Therefore, by calculating the surface integral of the squared *l*-th term of the series expansion, the energy contribution of the spatial frequency specified by the degree *l* and the radius $R_{4}$ to the potential on the outer surface can be determined. Due to this approach, the energy contribution can be determined independently of a triangulation of the model, defining surfaces and without discrete sampling of the potential on the outer surface.

We use the values determined in this way to validate Sphara on a spherical geometry.

To bridge the gap to practical applications, we have investigated five different sensor arrangements with 34, 104, 232, 462, 938 and 4000 electrodes, used for discrete sampling of the outer spherical surface, cf. Table 1. The sensors were arranged equidistantly on the head surface. The surface, defined by the sensor positions, is triangulated using the software toolbox distmesh (version from 2018, https://github.com/ionhandshaker/distmesh) [33]. The distances between the electrodes of the first five arrangements correspond to real EEG systems with 21, 64, 128, 256 and 512 electrodes [34,35]. We added the 4000 electrode arrangement to enable a high-resolution discrete spatial Fourier analysis in the sensor space. For each triangulation, the mean and maximum $h_{\max}$ edge lengths were determined, along with the corresponding spatial Nyquist wavelength $\lambda_{\mathrm{Nyquist}}$ and frequency $\omega_{\mathrm{Nyquist}}$, cf. Equation (13).

A total of 7700 unit current source positions are used in the model. For the sources outside the center, each 100 of these 7700 positions are arranged on 76 concentric spherical surfaces with radii of 1 mm to 76 mm. The positions are equally distributed on each of these concentric spherical surfaces, see also Figure 2b.

We performed the simulations using both radially and tangentially oriented dipoles. The radial dipoles are directed away from the center of the concentric spherical shells, cf. Figure 2c. The direction of tangential dipoles is randomly chosen in the plane orthogonal to the radial dipoles, cf. Figure 2d. For the sources located in the center of the concentric spherical surfaces (radius of 0 mm), it is not possible to distinguish between radial and tangential orientations. To these 100 central sources, random directions are assigned. To solve the forward problem for the spherical head model, we used a semi-analytical approach [32] implemented using the Fieldtrip toolbox [31].

### 3.2. Realistic Head Model Based on BEM

**Figure 3:**
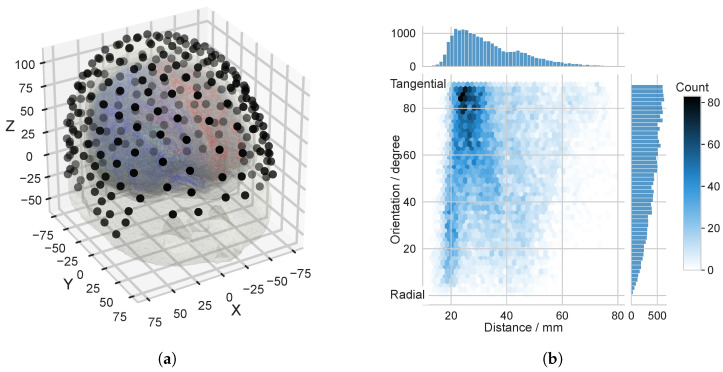
Realistic head model: (**a**) BEM model, consisting of five different compartments. The source positions are marked with blue and red dots (right and left hemisphere, respectively). For the area covered by the 256-channel EEG sensor setup (black dots), spatial frequency analysis using Sphara was performed. The units on the axes are mm. (**b**) Joint histogram of depth and orientation of the sources used in the head model: Depth is given as the distance from the sources to the nearest point on the scalp surface. Orientation is the angle between the dipole moment and the vector connecting the source position and the nearest point on the inner skull surface. The orientation is within the range from 0° to 90°, 0° corresponds to a radial orientation of the source.

For the analysis of the frequency response of the realistic head model, we use a BEM model, which is based on an example dataset from the SimNIBS project [36]. The model consists of the outer boundaries of gray matter (pial) and cerebellum, the inner and outer boundaries of the skull (inner skull, outer skull), and the outer scalp boundary (skin), see Figure 3a. Five different compartments are described by the five boundary surfaces: the brain (including white and gray matter), the cerebellum, the CSF, the skull, and the scalp.

The following conductivities are assigned to the individual compartments of the model: brain (white and gray matter), scalp and cerebellum 0.33 Sm^−1^, CSF 1.79 Sm^−1^, and skull 0.0066 Sm^−1^. The quantitative information on the triangular meshes used in the realistic head model can be found in Table 2.

For both the left and the right hemispheres of the brain, 10,001 dipoles are used for the forward computation. The source meshes are extracted from the white matter surface, i.e., the boundary of white and gray brain matter [25]. The unit strength dipoles are normally oriented with respect to the surface of the white matter. The distributions of depth and orientation of the sources used in the realistic head model are shown in Figure 3b. The depth is given as the distance from the source to the nearest point on the scalp surface. The orientation is the angle between the source moment and the vector, which connects the source position and the next point on the inner skull surface [37]. The range of values of the orientation is between 0 and π/2. At a distance of 20 mm or less from the surface of the head, quasi-radial dipoles (less than 30°) dominate, reflecting the dominance of gyri at the interface between the brain and the skull. Tangential orientations become increasingly frequent at greater depths (≈25 mm), corresponding to the anatomical location of the sulcal walls [37].

The Galerkin BEM approach was used for the forward calculation [25,38,39]. It was performed with the Helsinki BEM library [40,41].

We projected an equidistant EEG sensor setup with 256 channels onto the triangulated surface representing the scalp, and we determined the subset of the scalp triangle mesh covered by the EEG sensor system. We determined the Sphara basis functions for this subset of the triangle mesh, consisting of 2152 vertices and 4189 triangles. The analysis of the spatial frequency response of the realistic head model was performed using this Sphara base.

The full set of software toolboxes (SpharaPy (version 1.1.2), FieldTrip (version 20231220), Helsinki BEM library (version 160405)) was required for the validation study on spherical models, where Sphara was benchmarked against discrete spherical harmonics. For practical applications, however, only two inputs are required: (i) a triangulation of the EEG sensor system used, and (ii) a lead field matrix of the chosen head model. The lead field can be obtained using any preferred modeling approach, provided its format is known and the data can be imported. The subsequent spectral analysis can then be performed with SpharaPy alone.

## 4. Results

### 4.1. Validation Using a Spherical Head Model

To show the suitability of the Sphara approach, we compare the values of the spatial frequencies or spatial wavelengths determined numerically using the Sphara approach with the values of the spherical harmonics determined based on the Jeans relation. Secondly, we compare the results of the spatial spectral analysis (energy contributions for individual spatial frequencies) using Sphara and the discrete spherical harmonics. The semi-analytical approach serves as a reference, and the well-established approach of discrete spherical harmonics serves as a benchmark.

#### 4.1.1. Analysis of Spectral Properties of the Sphara Basis Functions

In the further course of our studies, we want to use Sphara bases for quantitative spatial spectral analyses. Therefore, it is necessary to estimate the degree of accuracy that we can achieve with the new Sphara approach using a given number of spatial sampling points. We investigate this issue by comparing the spatial frequencies determined using the Sphara approach for triangulated spherical surfaces with the analyticaly determined spatial frequencies of the spherical harmonics approach.

**Figure 4:**
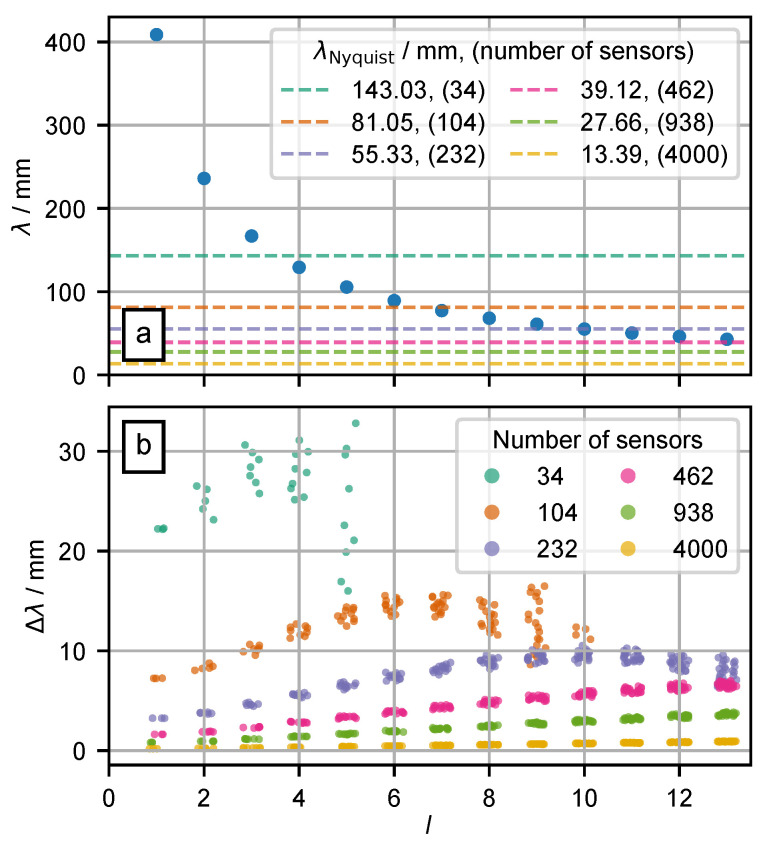
The wavelength λ calculated from the degree *l* of the (associated) Legendre polynomials, the radius of the outer spherical surface, and the differences in wavelengths Δλ of Sphara basis functions. On the x-axes of the two figures, the degree *l* of the spherical harmonics is indicated. (**a**) The wavelengths $\lambda_{\mathrm{SH}}$ of the spherical harmonics for a sphere with radius $R_{4}=92\;mm$ (blue dots). Dashed horizontal lines mark the Nyquist-wavelength limits for various sensor counts; only spectral components with λ above these limits should be interpreted. (**b**) The deviations Δλ and the spread of the wavelengths of the Sphara basis functions referring to $\lambda_{\mathrm{SH}}$. The number of sensors (spatial sample points) used to calculate the Sphara bases is color-coded. Please note, to reduce overplotting in the dot representation, a low jitter was applied in the x-direction (*l* are discrete, integer values).

We have computed the equivalent Cartesian spatial wavelength $\lambda_{\mathrm{SH}}$ of spherical harmonics for a given degree *l* and radius *R* using Jeans rule, cf. Equation (9). The values $\lambda_{\mathrm{SH}}$ for the degrees l=1…16 and radius $R_{4}=92\;mm$, which are identical to the terms of the series expansions, are shown in Figure 4a and given in the second column of Table A1 in Appendix A. In order to be able to interpret quantitatively the values of the determined equivalent spatial Cartesian wavelengths, the circumference of the spherical surface with radius R=92 mm is 578 mm. The basis function of degree 0 corresponds to the spatial direct component (DC) with an infinite wavelength and is not displayed.

We determine the Sphara bases for the six spatial samplings described in Section 3.1. The spatial wavelengths of the Sphara basis functions *λ*_Sphara_ corresponding to a certain degree *l* of spherical harmonics are computed according to Equation (4). The differences between the spatial wavelengths of the spherical harmonics and Sphara basis functions, as well as the spread of the wavelengths of the Sphara basis functions, are visualized in Figure 4b and shown in Table A1 in Appendix A.

The wavelengths of the Sphara basis functions are systematically smaller than those of the spherical harmonics. There is one main reason for this phenomenon: If a spherical surface is approximated by a triangular mesh, as in the Sphara approach, then only the vertices of the mesh (positions of the sensors) are located on the spherical surface. The triangular surfaces spanned by the sample points are inside the spherical surface. The wavelength of the Sphara basis functions is measured as the arc length along this polyhedron surface, which is always shorter than the arc length on the surrounding sphere.

Furthermore, an increase in the spread of the wavelengths determined with the Sphara approach can be observed as a function of degree *l*, cf. Figure 4b. This is stronger for sampling schemes with few spatial sampling points (34 and 104 spatial samples). The increase of this spread can be explained by the fact that the sampling density is not sufficient to represent the basis functions with the corresponding higher spatial frequencies.

By refining the triangular mesh and increasing the number of spatial sample points (number of sensors) to calculate the Sphara basis functions, the divergence between the wavelengths of spherical harmonics and Sphara decreases. Furthermore, the spread of the spatial wavelengths of the Sphara basis functions, which can be assigned to a single degree *l* of the spherical harmonics basis functions, also decreases, cf. Table A1 in Appendix A and Figure 4b.

#### 4.1.2. Analysis Using the Semi-Analytical Solution of the Spherical Forward Model

**Figure 5:**
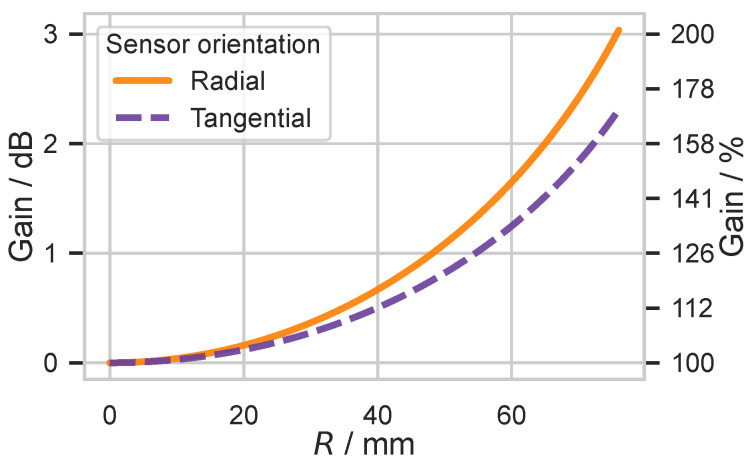
The energy gain of the potential distribution depending on the depth and the orientation of the unit sources. Reference (0 dB, 100%) is the unit source in the center of the concentric spheres. The orientation (radial, tangential) of the simulated unit sources is color-coded.

In the next step, we analyze the spatial spectral properties of the Legendre terms of the series expansion, which was used to solve the forward problem for the spherical head model in a semi-analytical way. The semi-analytical approach has the beneficial property that both the energy of the potential on the outer spherical surface and the energy contributions of the individual spatial frequency components can be determined without spatial sampling of the outer spherical surface and without triangulation of the concentric spherical surfaces of the head model. We analyzed the energy of the potential distribution on the head surface, which is caused by sources located at different depths. For each of the 77 source depths, both tangential and radial unit sources are simulated. In the further analysis steps of the spherical head model, we use the energy of the potential distributions on the scalp surface $R_{4}$, caused by a source at the center as a reference (0 dB or 100%), and we represent the energy contributions of the other sources relative to this. This allows for both the comparison of the power of potential distributions of sources of different depths as well as between radial and tangential sources. The results of this simulation are shown in Figure 5.

Compared to a central source, a near-surface (R=76 mm) radial source generates a potential with more than double the energy, and a near-surface tangential source generates a potential with 1.7 times the energy (cf. Figure 5), which also corresponds to the findings of [37].

**Figure 6:**
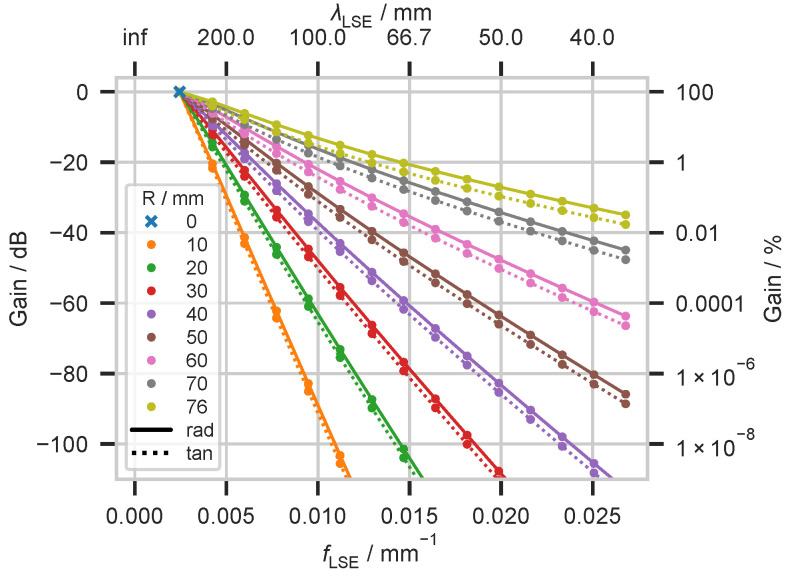
The amount of energy that terms of the series expansion of the semi-analytical forward model with a certain spatial frequency (specified by the degree *l*) contribute to the energy of the potential distribution. Reference (0 dB, 100%) is the energy contribution of the unit source in the center of the concentric spheres. The analyses for a unit source at each of the nine different depths are shown. The depth is given as the distance *R* from the center of the concentric head model. For better clarity, the individual points corresponding to a particular depth are connected by lines. Solid lines mark radial sources, and dotted lines mark tangential sources. On the two x-axes, both the spatial frequency and the spatial wavelength are indicated. On the y-axes, the relative energy contribution is given in dB as a percent.

**Figure A1:**
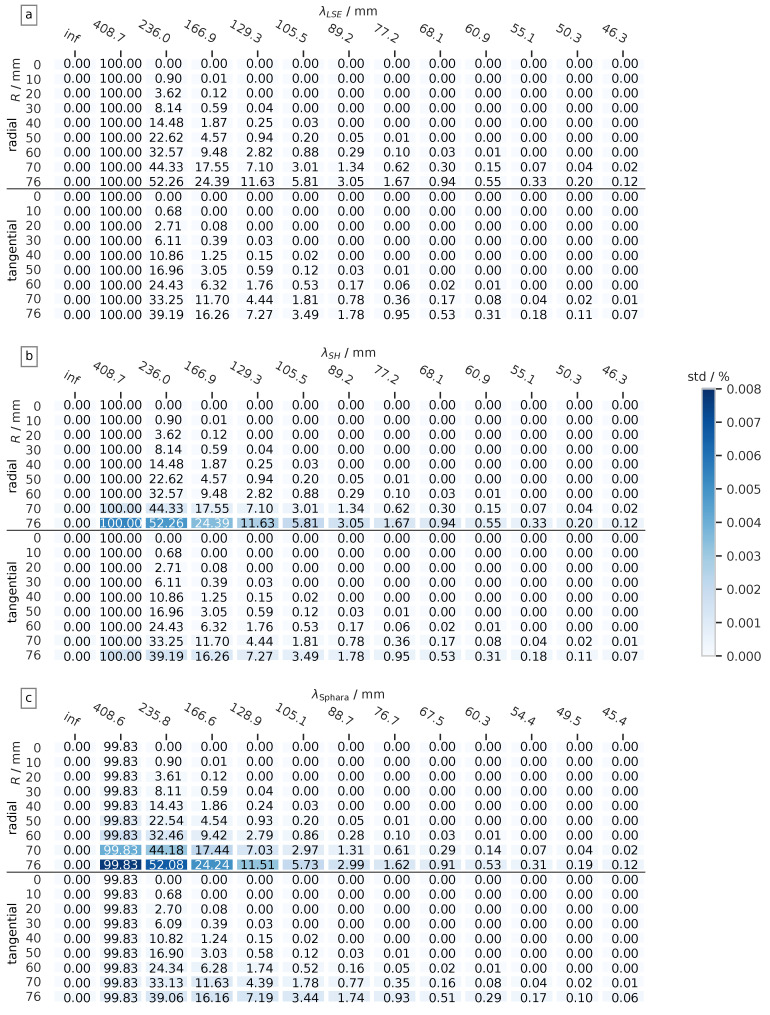
Spherical model: The percentage energy contributions of the individual terms of the series expansion of the semi-analytical approach (**a**), the discrete spherical harmonics (**b**), and the Sphara basis functions (**c**). The first line of the tables contains the spatial wavelengths $\lambda_{LSE}$, $\lambda_{SH}$, and $\lambda_{Sphara}$. In the first column of the tables, the orientation of the sources is given; in the second column, the distance $R_{\mathrm{source}}$ from the center of the concentric model is given. The percentage values in the tables (100%) is the energy contribution of the unit source in the center of the concentric spheres, see also Figure 5. The values shown for the percentage contributions are rounded to two decimal places. The values shown for the percentage contributions of spherical harmonics and Sphara are averaged values of 100 simulations for each source depth and rounded to two decimal places. The standard deviation is color coded in the background color. If the values in (**a**) are summed up row by row, one obtains the values shown in Figure 5.

Subsequently, we analyzed the energy contributions of the individual terms of the series expansion to the total energy of the potential evoked by dipoles at selected depths. Using the degree *l* of the Legendre polynomial and the radius $R_{4}$ of the outer spherical surface, a spatial frequency and thus a spatial wavelength can be assigned to each term of the series expansion; see also the first row of Figure A1a. We have examined terms up to degree l=15. The results of the analysis are shown in Figure 6 and Figure A1a in the Appendix A.

For a model consisting of concentric spheres, there is no energy contribution to the potential by the spatial DC component for dipoles from any depth. The energy contribution of the spatially low-frequency term subsequent to the DC component (degree l=1) is the same for sources of all depths, regardless of their orientation. For sources that are located in the center of the spherical head model, the term with degree l=1 of the series expansion exclusively contributes to the energy of the potential on the outer spherical surface. In Figure 6, it is marked by a blue cross. For sources located outside the center of the concentric model, further energy contributions from spatially higher-frequency terms are necessary to represent the potential distribution on the outer spherical shell. The energy gain in the potential, which can be observed for sources closer to the surface (compare Figure 5), can be explained by the additional energy contribution of the spatially higher-frequency terms, see Figure 6. Summing up the values in the rows of Figure A1a in Appendix A gives the values shown in Figure 5.

**Figure 7:**
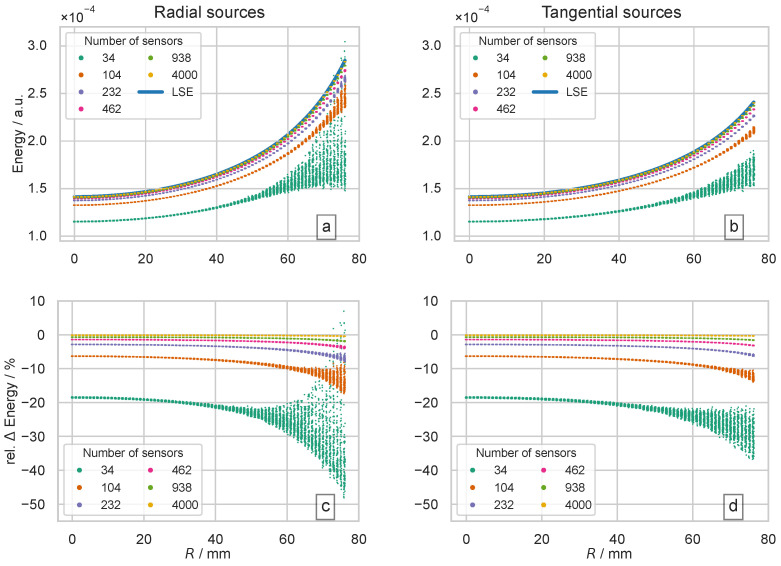
The energy of the potential distributions caused by the unit sources at different depths for the spherical head model. The depth of the sources is given as a the radius *R*. The signal energy was determined using six different sensor setups with different numbers of sensors; these are color-coded. The upper two subfigures (**a**) and (**b**) show the energy of the potential distributions captured by the different spatial samplings, while the lower two subfigures (**c**) and (**d**) show the relative deviations of the discrete spatial samplings from the semi-analytical approach. Simulations were performed separately for radial (**a**) and (**c**) and tangential sources (**b**) and (**d**). In each case, 100 unit sources are equally distributed on spherical surfaces with radii of 0 mm to 76 mm (step size 1 mm). For sources in the center, it is not possible to distinguish between radial and tangential orientations. At this position, 100 sources with random orientations are used for the simulation. For comparison, the energy determined by the analysis of the series expansion of the semi-analytical concentric head model is also indicated by a blue line (labeled by LSE).

So far, we have investigated the continuous properties of the semi-analytical solution of the forward problem for spherical head models. We now proceed to the discrete sampling of the outer spherical surface. In the next step, we investigate to what extent the spatial samplings of the outer spherical shell with different densities are suitable to adequately capture the potential distribution. Using the semi-analytical model, this aspect can also be analyzed independently of the triangulation of the individual layers of the concentric head model. To achieve this, radially and tangentially oriented sources were simulated separately. In each case, 100 unit sources are equally distributed on spherical surfaces with radii 0 mm to 76 mm and a step size of 1 mm. The energy of the potential distribution on the outer layer of the spherical head model was determined using the scalar product, as defined in Equation (3). The results of these simulations are shown in Figure 7.

The upper two subfigures, Figure 7a,b, show the energy of the potential distributions captured by the different spatial samplings. The lower two subfigures, Figure 7c,d show the relative deviations of the different discrete spatial samplings from the semi-analytical approach. The discrete spatial sampling systematically underestimates the energy contribution of the simulated potential distribution on the outer sphere surface compared to the semi-analytical approach, cf. Figure 7. The coarser the spatial sampling, the larger this underestimation of the energy of the potential distribution. The spread of the captured energy is noticeable, which occurs increasingly with coarse spatial samplings (34 and 104 sensors) and increasingly for near-surface sources, cf. Figure 7c,d. The closer the sources are to the outer surface of the model, the more spatially high-frequency components contribute to the shape of the potential distribution. A quantitative analysis of this phenomenon can be found in Figure A1a in Appendix A. The spatial sampling rates of the coarse sensor setups (34 and 104 sensors) are not sufficient to adequately capture the spatial high-frequency components, see also the penultimate paragraph of Section 2.2.

#### 4.1.3. Analysis of the Spatial Frequency Response of the Spherical Head Model Using Discrete Spherical Harmonics

**Figure 8:**
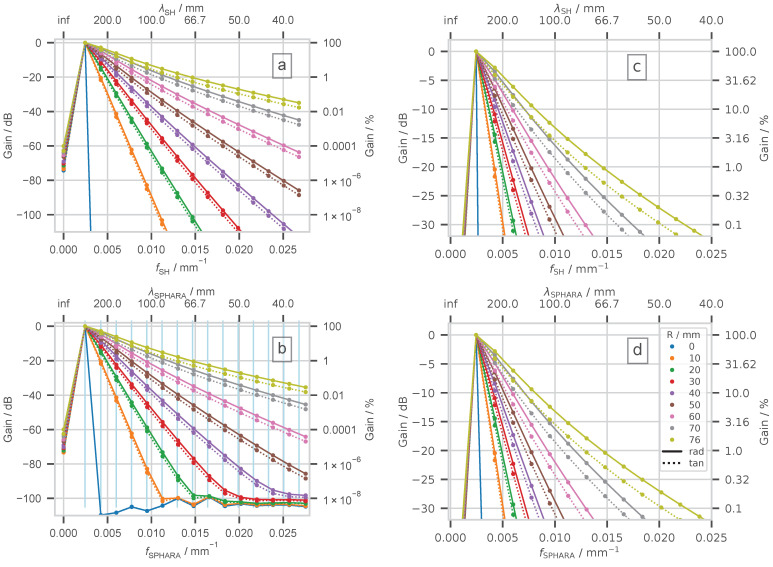
The amount of energy that basis functions with a certain spatial frequency contribute to the energy of the potential distribution: Reference (0 dB, 100%) is the mean value of the energy of the potential distributions of 100 unit sources in the center of the concentric spheres. The analysis was performed only at the frequencies marked by dots. For clarity, the individual points corresponding to a particular depth are connected by lines. The analyses for unit sources are shown for nine different depths *R*. Solid lines mark radial sources, and dotted lines mark tangential sources. On the two x-axes, both the spatial frequency and the wavelength are indicated. On the y-axes, the relative energy contribution is given in dB and in percent. The two upper subfigures (**a**) and (**c**) show the results of the discrete spherical harmonic analysis, and the two lower subfigures (**b**) and (**d**) show the results of the Sphara analysis. The two subfigures on the right (**c**) and (**d**) contain enlarged cutouts of the subfigures on the left (**a**) and (**b**) respectively, showing values that are of higher practical relevance. The color coding is identical for all subplots.

Next, we use the discrete spherical harmonics analysis, a well-established method of Fourier analysis for data defined on spherical surfaces, to analyze the spatial frequency response of spherical head models. The results of the discrete spherical harmonic analysis serve as a benchmark for the later analysis of the spatial frequency response using Sphara. To show the very good agreement of both approaches for spherical head models, only the high-resolution sampling of the outer spherical surface with 4000 spatial samples was used. The results of this investigation are shown in Figure 8a,c. A source in the center of the concentric head model serves as a reference for the energy data. Theoretically, the energy of the potential caused by this source should be exclusively reflected in the spatially lowest frequency following the DC component and correspond to 100% or 0 dB in the figures. The spatial frequencies of the spherical harmonics correspond to those of the semi-analytical model because they are calculated on the same basis, using the degree *l* of the Legendre polynomials and the radius *R* of the outer spherical surface of the head model. Also, the energy contributions of the individual spatial frequency components estimated based on the discrete spherical harmonic analysis correspond to a large extent to the values from the simulation to at least two decimal places, cf. Figure 6 and Figure 8a as well as Figure A1a,b.

Contrary to expectations, the discrete spherical harmonics analysis of the spherical surface model of the head shows a energy contribution for the spatial DC component, cf. Figure 6 and Figure 8a. However, these contributions are for all sources below −60 dB in comparison to the total energy of a unit source at the center of the spherical surface model, and they have no practical relevance. These are likely caused by numerical effects.

#### 4.1.4. Analysis of the Spatial Frequency Response of the Spherical Head Model Using Sphara

Next, we examined the frequency response of the spherical head model utilizing the Sphara approach. We determined the basis functions using the FEM discretization of the Laplace–Beltrami operator for the spatial sampling scheme with 4000 sampling points, cf. Table 1. In Section 4.1.1, we quantified the deviation of the wavelength determined by the Sphara approach from the reference values, see Figure 4b and the right column in Table A1. We estimated the energy contributions of the Sphara basis functions for the 7700 radial and tangential sources used in the model, and we averaged 100 energy contributions per Sphara basis function from sources of the same depth. These averaged values of the 16 low-frequency spatial spectral components, which also provide the highest energy contribution to the potential on the outer spherical surface, are shown in the lower part of Figure 8b,d and in Figure A1c. The standard deviations of all averaged values shown in Figure A1c are smaller than 0.008%. Comparing these values with the reference values shown in Figure A1a, it can be seen that the energy contributions are systematically slightly underestimated. The underestimation increases marginally for near-surface sources, but for these sources, it is in the per mille range with respect to the reference value. Very small energy contributions of the Sphara coefficients (<−90 dB) are not determined correctly, see Figure 8b. These deviations are of such a small order that they have no relevance for practical applications. They are also due to numerical effects.

### 4.2. Application to Realistic Head Model

**Figure 9:**
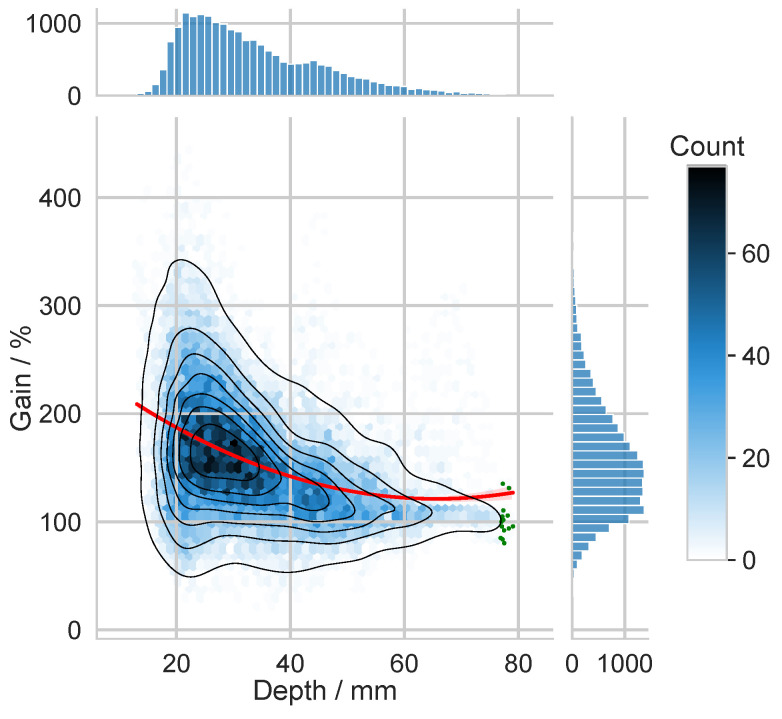
Joint histogram of the distance of the 20,002 sources from the scalp of both hemispheres and the energy of the potential distribution on the scalp covered by the EEG sensor system. Reference (0 dB, 100%) is the median of the energy emitted by the 15 sources situated most distant from the scalp (marked by green dots). The red line shows a fitted second-order polynomial regression curve, illustrating the overall trend between source depth and the energy contribution.

**Figure 10:**
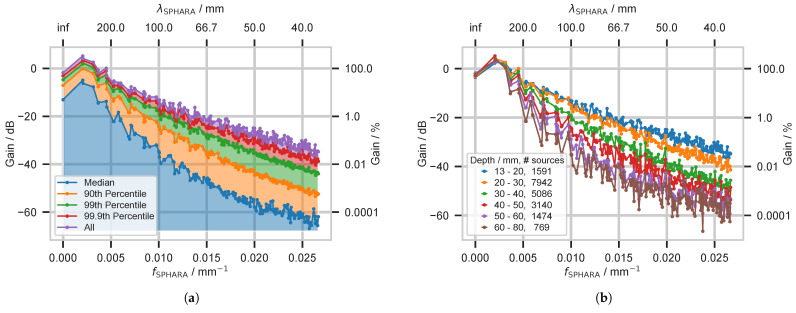
Spatial frequency components of the realistic head model: (**a**) The energy contributions to the spatial frequency components of the analyzed potential for all considered sources. The median, 90th, 99th, and 99.9th percentiles of the energy contribution of all the sources is shown. Reference (0 dB, 100%) is the median of the energy emitted by the 15 sources situated most distant from the scalp. (**b**) Analysis of the frequency response of sources from different depths. The sources were divided into six depth groups; the depths and and the number of sources in the group are shown in the legend. The lines in the diagram indicate the maximum energy contribution for each depth range for the corresponding spatial frequency.

Finally, we analyzed the realistic BEM model of the human head, which we described in Section 3.2. First, we investigated the dependence of the distance of the sources from the head surface and the energy of the potential distribution on the head surface covered by the EEG sensor system. The results of this analysis are shown in Figure 9 as a joint histogram using the absolute frequencies of these two considered features. In the joint histogram, the energy is given in terms of the median of the energy of the potential of the area of the scalp covered by the EEG system emitted by the 15 sources situated most distant from the head surface. This median value corresponds to 0 dB or 100%. This reference is also used for the presentation of further results in Figure 10 and in Table A2 in the Appendix A. The 15 selected reference sources are marked by green dots in Figure 9. In the marginal histograms, the distributions of depth and the energy contributions of the sources are also shown separately.

For the Sphara analysis, we applied the Sphara decomposition to the spatial potential distribution on the scalp for each individual source of the head model, and we determined the energy contributions of the Sphara coefficients. All energy contributions for each spatial frequency were sorted according to their magnitudes. In Figure 10a, the median, 90th, 99th, 99.9th percentile, and the maximum energy contribution of all sources and all spatial frequency components to the potential on the head surface are shown. Thus, the energy distributions of all sources to the spatial frequencies are illustrated. Considering all sources, the violet line indicates the maximum energy contribution that one of these sources can provide for a given spatial frequency. Due to the fact that in the realistic head model, the analysis of the spatial frequency response only includes a part of the outer head surface, the spatial DC component also provides a considerable energy contribution. In contrast to the spherical head model, the spatial low-frequency spectral components following DC in the realistic head model have an energy contribution larger than 100% relative to the reference.

The analysis of the spherical head model shows that the frequency response depends considerably on the depth of the sources. To investigate this aspect for the realistic head model, we divided the sources into six groups according to their depth [42]. The limits of the depth ranges and the number of sources in these groups can be seen in the legend in Figure 10b. For each source in the selected depth range, we decomposed the caused potential distribution using Sphara and determined the energy contributions for the individual spatial frequency components. In Figure 10b, we show the maximum energy contribution of all sources for each depth range for the particular spatial frequency. Potential distributions caused by sources near the surface contain a larger energy contribution of spatially high-frequency components. In frequency components with a spatial wavelength of 50 mm, the largest energy contribution of a near-surface source is 0.32% compared to the reference, and the contribution decreases further for frequency components with lower spatial wavelengths, cf. Figure 10b and Table A2.

## 5. Discussion

### 5.1. Verification of Sphara Wavelength Estimates

Spatial Fourier bases tailored to arbitrary sensor layouts can be generated with the Sphara approach. When the method was evaluated on the spherical reference model, the spatial wavelengths obtained from the discrete Sphara basis were found to be *systematically shorter* than the analytical wavelengths of the corresponding spherical harmonics computed on the same surface (cf. Figure 4b). This underestimation arises because every vertex lies on the spherical shell, whereas the triangular facets that connect them sag slightly inside the surface; consequently, the geodesic paths on which Sphara measures wavelength are reduced.

The magnitude of the bias was shown to depend on the sampling density: for the two densest meshes (938 and 4000 vertices), the mean error remained in the low single-digit-percent range, whereas at 104 and 34 vertices, the error exceeded 10% and was accompanied by a markedly larger dispersion, particularly for the highest spatial orders that were still representable. Accordingly, when quantitative results are interpreted, it should be kept in mind that Sphara provides a slightly conservative estimate of wavelength, and that the bias and its variance grow as vertex spacing increases. In practical terms, meshes with fewer than roughly 100 sampling points ∼25 mm electrode spacing on an adult scalp) should therefore be considered below the reliable threshold for accurate wavelength quantification.

### 5.2. Validation of Sphara Energy Estimates and Spatial Power Spectra

For the concentric four-shell head model, the forward problem can be expressed as a series of Legendre terms, cf. Equations (14)–(17). Based on the radius $R_{4}$ and the degree *l*, which are used in these terms, a spatial wavelength and frequency can be assigned to each term, to which this term exclusively contributes. By squaring the Legendre terms and integrating on the sphere surface, one can exactly determine its energy contribution.

To create a reference, the total scalp-potential energy generated by unit dipoles was evaluated as a function of source depth and orientation. As expected, energy decreased monotonically with depth, and radial generators produced more energy than tangential generators at identical depths, cf. Figure 5. Spectral decomposition showed that sources in the center of the sphere contributed exclusively to the first alternating component above the direct current component, while sources closer to the surface contained increasingly more energy in spatially higher-frequency components, cf. Figure 6 and Figure A1a. Although the absolute energy varied with depth and orientation, the amplitude of the first alternating component remained constant across all source locations; the additional energy observed for near-surface sources was carried solely by these spatially higher-frequency components.

In practical EEG, the potential is sampled only at discrete scalp locations. To assess how such sampling affects the recovered energy, sensor layouts mimicking common clinical and high-density systems, as well as an ultra-dense layout of 4000 points, were simulated, cf. Table 1. The energy contained in each sampled distribution was computed with the discrete inner product defined in Equation (3). It was found that every finite layout underestimated the true energy, with the bias increasing as the mean inter-electrode distance grew. For the coarsest grid (34 samples, ≈60 mm spacing), the total energy was underestimated by roughly 20% (cf. Figure 7). The variance of the captured energy also increased with decreasing sample density, especially for sources located at radii > 50 mm (34 samples) or >70 mm. These trends can be explained by the higher-frequency components generated by near-surface, especially radial, dipoles; such components are not adequately represented by the spatially high-frequency basis function of the coarse grids, cf. Figure 4 and Figure 8. This finding is also consistent with the observations of Haueisen et al. [37].

The underestimation of energy observed in low-density electrode setups is directly linked to the spatial Nyquist frequency $\omega_{\mathrm{Nyquist}}$ defined by the maximum edge length of the mesh. Near-surface, primarily radial sources generate potential distributions with significant energy contributions at spatial frequencies that can approach or exceed $\omega_{\mathrm{Nyquist}}$, particularly in low-density sensor configurations. Consequently, sparse systems such as the 10–20 layout (34 sensors in the spherical model), with a Nyquist wavelength of approximately 143 mm (cf. Table 1), are inherently incapable of capturing the full spatial energy spectrum of superficial gyral sources. Hence, in low-density EEG recordings, power analyses must be interpreted cautiously, particularly when studying activity generated by superficial cortical regions.

In summary, the analytical energy benchmark confirmed that Sphara partitions potential energy accurately when the scalp is densely sampled, but that low-density electrode setups introduce a systematic underestimation that becomes substantial once electrode spacing exceeds ∼35 mm. This finding complements the wavelength analysis in Section 5.1 and underscores the need for sufficiently dense sensor arrays when quantitative spatial energy or power-spectrum measures are required.

### 5.3. Comparison of the Spatial-Frequency Response Obtained with Discrete Spherical Harmonics and Sphara

The spatial power spectrum of the concentric four-shell head model was computed with both the discrete spherical harmonics and Sphara. A dense set of 4000 sampling points was employed on the outer spherical surface in order to suppress under-sampling bias and isolate algorithmic differences.

In each analysis, the direct-current (DC) component contributed less than −60 dB to the total energy (cf. Figure 8a,b). This negligible offset is attributed to numerical round-off and mesh interpolation rather than to a genuine physical component. Across the remaining spatial orders, the two methods agreed to within 0.2% in the worst case, and to better than 0.05% for the majority of orders. Noticeable deviations appeared only for energy levels below −80 dB, i.e., well below the range relevant for practical EEG analysis.

The discrete spherical harmonics spectrum reproduced the semi-analytical Legendre expansion to at least two decimal places (cf. Figure 6 and Figure A1a), whereas the Sphara spectrum exhibited marginally larger, but still sub-percent, deviations. These differences arise chiefly from the slight wavelength underestimation discussed in Section 5.1.

Although discrete spherical harmonics achieves marginally higher numerical accuracy on the ideal sphere, Sphara retains satisfactory fidelity while remaining applicable to arbitrarily shaped and non-uniformly sampled surfaces, as demonstrated in the subsequent BEM head analysis. Accordingly, Sphara can be regarded as a reliable and more generalizable alternative to discrete spherical harmonics when dense, uniform sampling cannot be guaranteed.

### 5.4. Estimation of the Spatial-Frequency Response of a Realistic Head Model with Sphara

Because an EEG sensor array samples only a portion of the scalp, the electric potential is acquired incompletely on realistic heads. In the present analysis, radial and tangential generators were not segregated because the energy captured by a given electrode setup depends both on the dipole orientation with respect to the inner skull and on its orientation relative to the sensor layout. The cortical sources considered here are not depth-uniform; most lie 20mm to 30mm beneath the scalp, cf. Figure 9. As expected, sources closer to the surface tend to yield greater scalp energy, yet the source-to-source variability is substantially larger than in the spherical model.

For practical applications such as spatial-filter design or data compression, the maximum energy observed at each spatial frequency across all sources is a key quantity. This upper envelope is depicted by the purple line in Figure 10a. The envelope decays approximately as $1/f^{\kappa }$ with increasing frequency. To illustrate the statistical spread, the median and selected percentiles are also shown. In the present head and source model, spectral components whose wavelengths are shorter than 50 mm contribute less than 0.32% of the total energy.

The depth dependence of the spectrum was examined separately, cf. Figure 10b. Near-surface dipoles were found to supply a larger fraction of their energy to high-frequency components, whereas deeper sources were dominated by low-frequency content. This behavior is consistent with the spherical benchmark and with earlier reports [37,42].

A pronounced DC component was observed in the realistic model. This is not an artefact of the algorithm but a consequence of performing the spectral analysis on an open surface that does not fully enclose the cortical generators. When quantitative EEG measures are interpreted, the spatial spectra of both the neural signal and the measurement noise should therefore be evaluated in parallel [43].

In this context, the spatial Nyquist frequency of a sensor setup serves as a critical benchmark for evaluating its suitability for quantifying power changes. Electrode setups with a low $\omega_{\mathrm{Nyquist}}$ relative to the highest cortical spatial frequencies, such as the 10–20 system, inherently lack the resolution to capture the energy contributions of superficial sources, especially those with predominantly radial orientations that produce spatially confined scalp potentials [37]. For studies targeting spatial power distributions or subtle power changes, such as those associated with cognitive states or localized cortical activations, EEG systems should be chosen with a Nyquist wavelength adapted to the expected source depth, orientation, and the acceptable margin of power estimation error. Specifically, a lower Nyquist wavelength is required to resolve the higher spatial frequencies of superficial radial sources, whereas deeper or tangential sources can be sufficiently represented with coarser sampling. Sensor density should thus be aligned with the spatial characteristics of the neural activity under investigation and the precision demanded by the analytical goals.

### 5.5. Comparative Evaluation with Existing Methods

To complement the state-of-the-art overview in the Introduction, we focus our comparison on the two paradigms historically used to quantify spatial low-pass properties of the human head: (i) discrete spherical harmonics and (ii) one-dimensional (1D) Fourier analysis of cross-sectional contours. We contrast these with Sphara, which generalizes spatial Fourier analysis to arbitrarily sampled surfaces via the Laplace–Beltrami eigenbasis of the scalp mesh.

Discrete Spherical Harmonics

SH provide a global spherical basis and are exact on spherical head models with appropriate quadrature; on realistic head’s they require mapping the scalp to a sphere and masking unsampled regions. The transform is unitary on the sphere and allows direct wavelength interpretation via the degree *l*. Main uncertainties arise from mapping distortions, sampling nonuniformity of the mapped grid, and window (mask) effects at excluded regions.

1D Contour/Slice Fourier

This approach analyzes spectra along selected cross-sectional contours through the head. It is intuitive and lightweight, but it relies on slice selection and projection from the 3D surface to 1D paths. Unitarity holds only along each chosen path, and wavelengths are defined along that path. Typical uncertainties include slice-selection bias, projection/boundary effects, and possible aliasing along contours.

Sphara

Sphara uses the eigenmodes of the discrete Laplace-Beltrami operator on the scalp mesh and operates directly on arbitrary triangulations, including irregular sensor layouts and realistic head geometries. The transform is unitary with respect to the FEM mass matrix *B* and offers a direct wavelength mapping, see Equation (4). Remaining uncertainties relate to mesh quality (e.g., the maximum edge length $h_{\max}$ and coverage).

On spherical validation with equidistant sampling, SH and Sphara yield comparable reconstructions at matched spatial bandwidths. For realistic heads and arbitrary sensor layouts, Sphara affords an energy-preserving decomposition on the measured surface without mapping or windowing, while 1D contour analyses remain useful as qualitative diagnostics but provide lower-dimensional views and should be interpreted accordingly.

### 5.6. Practical Implications for Sensor System Design and Analysis Pipelines

The systematic wavelength and energy biases documented above can be translated into specific recommendations for sensor layout. Based on the current simulations, it is recommended that scalp meshes with at least 128 electrodes (with a mean spacing of ≈25 mm) be used when quantitative spatial–spectral measures are required. At lower sensor densities, the underestimation of wavelength and energy exceeds the single-digit-percent tolerance established in Section 5.1. In practice, arrays with around 100–120 channels already capture most physiologically relevant spatial content, while denser layouts primarily improve robustness against noise and enhance reliability under non-ideal recording conditions.

This threshold aligns well with the empirical network-recovery plateaus reported for infant and adult EEG [8] and with the ’super-Nyquist’ benefits observed for very-high-density electrode setups [44]. Furthermore, optimal layouts derived from the generalized spatial–frequency framework of [7] converge on similar channel counts, indicating that the recommendation is robust across independent methodologies.

When planning an experiment, the expected source depth and the desired fraction of captured spatial energy should therefore guide sensor density. Deep sources are represented mainly by low spatial–frequency components and can be sampled adequately even with fewer electrodes. In contrast, sources close to the skull contribute substantially more energy and extend into higher spatial frequencies; capturing these components reliably requires denser electrode layouts. Taken together, these findings indicate that Sphara provides both a rigorous quantitative benchmark for spatial–frequency analysis and a practical framework for aligning EEG sensor design with experimental goals.

Because Sphara supplies geometry-adapted spatial basis functions together with their associated spatial wavelengths (derived from eigenvalues), objective metrics such as cumulative energy capture or eigenvalue roll-off can be computed automatically and used for sensor placement optimization or adaptive spatial filtering in real time. Inverse solvers that operate in geometry-aware harmonic bases may also benefit from an initial projection onto a truncated Sphara subspace. Examples include the anatomical-harmonics approach of [9] and the earlier Harmony method of [45], which reconstructs sources in a basis of spherical harmonics constrained by anatomy. In both cases, the dimensionality of the inverse problem could be reduced without sacrificing head-specific information, thereby accelerating computation while preserving accuracy. Together, these considerations demonstrate that Sphara not only quantifies spatial bandwidth but also provides practical tools for signal processing and for enhancing both forward and inverse EEG workflows.

In addition, our results offer a useful reference for emerging data-driven approaches that attempt to reconstruct high-resolution EEG from sparse or low-density recordings [10,11,46]. Related work has also demonstrated the use of convolutional neural networks to enhance EEG spatial resolution based on BEM-simulated data [46]. By quantifying the true spatial-frequency limits of the head, Sphara-based analyses can complement such AI and machine learning (ML) methods by providing physically meaningful priors and validation targets, ensuring that learned reconstructions remain biophysically plausible.

### 5.7. Limitations and Outlook

Several simplifying assumptions underlie the present study. First, isotropic conductivities were assigned to all compartments of the head, even though white matter, skull, and scalp are known to exhibit anisotropic properties. Inclusion of anisotropy in the forward model would therefore be desirable and should be examined in future work. Second, a standard BEM model comprising the cerebellum, the CSF, the skull, and the scalp was used. Recently, high-resolution BEM-FMM (BEM accelerated by the fast multipole method) head models have been introduced, which include seven compartments and especially the separation of gray and white matter, ref. [47], similar to FEM models [6]. Although we do not expect large differences in the outcome of the spatial spectra for standard and high-resolution models, studies are needed to confirm this assumption. Third, all simulations were performed on a single adult head; it remains to be verified whether the spatial--frequency characteristics reported here generalize to pediatric or geriatric populations, where head size, skull thickness, and tissue conductivities differ markedly.

Looking ahead, the Sphara basis could be incorporated directly into inverse solvers, replacing canonical Euclidean priors with geometry-adapted ones and potentially reducing computational cost. Real-time applications such as brain–computer interfaces might also benefit: by truncating the Sphara expansion to the energy-dominant modes identified in Section 5.4, low-latency spatial filtering and compressed sensing could be achieved without significant information loss. Advances along these lines are expected to further bridge the gap between theoretical spatial–spectral analysis and practical EEG signal processing.

Although the present work focuses on a fundamental, physics-based characterization of the human head’s spatial frequency response, the findings may also support future developments in ML. In particular, spectral priors derived from Sphara or from Nyquist-based considerations could be integrated as regularization terms in model-based ML approaches or as constraints within physics-informed neural networks, thereby combining biophysical plausibility with data-driven flexibility.

## Figures and Tables

**Table 1:** Sensor arrangements used for the forward simulations with the spherical head model: The numbers of vertices (sensors) and triangles are provided, as well as the mean and maximum triangle side lengths. The spatial Nyquist wavelength $\lambda_{\mathrm{Nyquist}}$ and frequency $\omega_{\mathrm{Nyquist}}$ are computed based on the maximum side length $h_{\max}$.

Real	Electrodes		Side Length		Nyquist Limit	
Electrode	on Spherical	Triangles,	$h_{\mathrm{mean}}\pm \mathrm{SD}$	$h_{\max}$	$\lambda_{\mathrm{Nyquist}}$	$\omega_{\mathrm{Nyquist}}$
System, No.	Surface, No.	No.	mm	mm	mm	mm^−1^
21	34	64	59.73±5.01	71.52	143.03	0.007
64	104	204	34.42±2.44	40.52	81.05	0.012
128	232	460	23.10±1.57	27.66	55.33	0.018
256	462	920	16.38±1.08	19.56	39.12	0.026
512	938	1872	11.50±0.75	13.83	27.66	0.036
–	4000	7996	5.57±0.36	6.69	13.39	0.075

**Table 2:** The five boundary surfaces used in the BEM model of the realistic head model: The surfaces used in the BEM model are specified by triangular meshes. The numbers of vertices and triangles are given, as well as mean value and standard deviation of the triangle side lengths.

Surface	No. Vertices	No. Triangles	Side Length Triangles/mm
Pial	12,501	24,998	4.0 ± 1.0
Cerebellum	999	1998	5.0 ± 1.0
Inner Skull	3801	7598	5.0 ± 1.0
Outer Skull	2901	5798	6.0 ± 1.2
Skin	3296	6600	7.0 ± 1.4

## Data Availability

All relevant data will be available from the corresponding authors upon reasonable request.

## Appendix A. More Comprehensive, Quantitative Results

**Table A1:** Mean value and standard deviation of the differences in spatial wavelengths between spherical harmonics and the Sphara basis functions: In the first and second columns, the degree *l* and the spatial wavelength λ of the spherical harmonics are given. Columns 3 to 8 contain the mean values and standard deviations of the wavelength differences Δλ between spherical harmonics and Sphara basis functions sampled with 34 to 4000 spatial samples. The relative deviation (in percent) from the spatial wavelength of the spherical harmonics is given in brackets.

*l*	$\lambda_{\mathrm{SH}}$/mm	Δ*λ*_Sphara,43_/mm	Δ*λ*_Sphara,104_/mm	Δ*λ*_Sphara,232_/mm	Δ*λ*_Sphara,462_/mm	Δ*λ*_Sphara,938_/mm	Δ*λ*_Sphara,4000_/mm
1	408.7	22.2 ± 0.1 (5.4%)	7.2 ± 0.0 (1.8%)	3.2 ± 0.0 (0.8%)	1.6 ± 0.0 (0.4%)	0.8 ± 0.0 (0.2%)	0.2 ± 0.0 (0.0%)
2	236.0	25.0 ± 1.4 (10.6%)	8.3 ± 0.3 (3.5%)	3.8 ± 0.1 (1.6%)	1.9 ± 0.0 (0.8%)	0.9 ± 0.0 (0.4%)	0.2 ± 0.0 (0.1%)
3	166.9	28.3 ± 1.7 (17.0%)	10.2 ± 0.4 (6.1%)	4.6 ± 0.1 (2.8%)	2.3 ± 0.0 (1.4%)	1.2 ± 0.0 (0.7%)	0.3 ± 0.0 (0.2%)
4	129.3	27.8 ± 2.1 (21.5%)	12.0 ± 0.5 (9.3%)	5.6 ± 0.2 (4.3%)	2.8 ± 0.1 (2.2%)	1.4 ± 0.0 (1.1%)	0.3 ± 0.0 (0.3%)
5	105.5	23.9 ± 6.1 (22.7%)	13.6 ± 0.6 (12.9%)	6.5 ± 0.2 (6.2%)	3.3 ± 0.1 (3.2%)	1.7 ± 0.0 (1.6%)	0.4 ± 0.0 (0.4%)
6	89.2		14.6 ± 0.6 (16.3%)	7.4 ± 0.3 (8.3%)	3.8 ± 0.1 (4.3%)	1.9 ± 0.0 (2.2%)	0.5 ± 0.0 (0.5%)
7	77.2		14.6 ± 0.7 (18.9%)	8.2 ± 0.3 (10.6%)	4.3 ± 0.1 (5.6%)	2.2 ± 0.1 (2.8%)	0.5 ± 0.0 (0.7%)
8	68.1		13.2 ± 1.1 (19.4%)	8.9 ± 0.4 (13.0%)	4.8 ± 0.2 (7.1%)	2.4 ± 0.1 (3.6%)	0.6 ± 0.0 (0.9%)
9	60.9		12.8 ± 2.5 (21.0%)	9.4 ± 0.4 (15.4%)	5.3 ± 0.2 (8.6%)	2.7 ± 0.1 (4.4%)	0.6 ± 0.0 (1.1%)
10	55.1		11.8 ± 0.6 (21.5%)	9.6 ± 0.4 (17.4%)	5.7 ± 0.2 (10.3%)	2.9 ± 0.1 (5.4%)	0.7 ± 0.0 (1.3%)
11	50.3			9.4 ± 0.5 (18.7%)	6.0 ± 0.2 (12.0%)	3.2 ± 0.1 (6.3%)	0.8 ± 0.0 (1.5%)
12	46.3			8.7 ± 0.5 (18.9%)	6.3 ± 0.2 (13.7%)	3.4 ± 0.1 (7.4%)	0.8 ± 0.0 (1.8%)
13	42.8			7.8 ± 0.9 (18.3%)	6.6 ± 0.2 (15.3%)	3.6 ± 0.1 (8.5%)	0.9 ± 0.0 (2.1%)

**Table A2:** Analysis of the frequency response of sources from different depths for the realistic BEM head model. The sources were divided into six depth groups; the depths and and the number of sources in the group are shown in the first two columns. Ten spatial wavelengths were selected, for which the corresponding energy contributions are specified; see the first line. The energy contributions are given relative to the median of the energy of the potentials generated by the 15 deepest sources. In each case, the value for the source from the depth range with the largest energy contribution for the respective spatial wavelength is given.

*λ*_Sphara_/mm		*∞*	486.8	317.3	273.0	221.8	190.2	146.2	100.0	75.4	50.0
Depth Range/mm	#Sources	Gain/%									
13–20	1591	43.82	169.07	165.26	86.43	60.86	23.90	12.08	4.27	0.62	0.32
20–30	7942	47.61	218.76	181.30	52.82	101.83	18.06	7.98	3.20	0.38	0.07
30–40	5086	64.05	214.32	136.44	34.81	78.07	9.67	4.08	1.25	0.13	0.01
40–50	3140	52.67	331.58	106.53	24.64	53.50	5.31	1.03	0.38	0.03	0.00
50–60	1474	65.79	181.91	118.46	23.87	32.08	2.48	0.34	0.13	0.01	0.00
60–80	769	57.05	216.31	108.37	9.50	14.56	0.70	0.10	0.03	0.00	0.00
